# Research on optimal strategy of different fire rescue tasks based on oxygen consumption

**DOI:** 10.3389/fphys.2025.1548031

**Published:** 2025-03-24

**Authors:** Jinyong Tang, Xinxin Zhang, Ziwen Wang, Tiehuai Liang, Weiguo Liu

**Affiliations:** ^1^ College of Physical Education and Health, Guangxi Normal University, Guilin, China; ^2^ School of Outdoor Sports, Guilin Tourism University, Guilin, China

**Keywords:** firefighter, rescue strategy, strength and conditioning level, load-carrying technique, oxygen consumption

## Abstract

**Objective:**

The development of effective rescue strategies is critical for enhancing rescue operations and ensuring firefighter safety. However, limited attention has been given to the exploration of rational rescue strategies in practice, particularly with regard to oxygen consumption. Therefore, this study aimed to identify the optimal rescue strategy by analyzing oxygen consumption across different rescue tasks.

**Methods:**

Sixty male firefighters from the Guilin Fire and Rescue Detachment participated in the study. Their oxygen consumption was measured during the completion of running on flat ground and while running up and down three flights of stairs.

**Results:**

The results found that firefighters with excellent strength and conditioning levels, those carrying a 10 kg load, or those employing the shoulder-carrying technique had less oxygen consumption. Hand-carrying for a 10 kg load when running up or downstairs and shoulder-carrying for 20 and 30 kg loads while running on the ground resulted in lower oxygen consumption. Additionally, firefighters with excellent strength performance when running with 10 and 20 kg loads or those with excellent speed while running on the ground exhibited decreased oxygen consumption.

**Conclusion:**

The current study suggests that firefighters with excellent strength performance are more suited for upstairs rescue tasks, while those with excellent speed performance are better suited for tasks on ground. It is recommended that medium to large loads be carried using the shoulder-carrying technique, and smaller loads be hand-carried when running up or down stairs. Overall, customizing rescue strategies based on firefighters’ strength and conditioning, load characteristics, techniques, and specific task requirements is crucial for improving efficiency and reducing risks in rescue operations.

## Introduction

Given the unique working environment and specific occupational demands of firefighters, they are often required to utilize heavy equipment to carry out several complex and diverse rescue tasks, such as climbing stairs and carrying materials (Lavender et al., 2014). The intricacy of these rescue tasks, coupled with their high work intensity, has long presented significant occupational safety threats to firefighters (Zhang et al., 2024; Bai et al., 2021; Ras et al., 2024). Enhancing firefighters’ rescue capabilities has been a focal point for researchers, as their proficiency in executing rescue tasks and their ability to fully leverage their skills directly impact task efficiency (Weidinger, 2022; Fyock-Martin et al., 2020), thereby indirectly affecting national and public safety (Heydari et al., 2022; Dowdall-Thomae et al., 2012). Addressing how to enhance the efficiency of rescue tasks while concurrently mitigating safety and injury risks associated with rescue operations represents an urgent scientific challenge within firefighting and rescue (Lee et al., 2020; Chae and Seung, 2022).

The complexity and diversity inherent in rescue scenarios necessitate elevated standards for firefighters’ strength and conditioning levels and the rational application of rescue strategies (Zhang et al., 2024). This need is particularly pronounced in specialized situations where oxygen supply represents a limiting factor (Lee et al., 2013). In such contexts, the efficient utilization of oxygen becomes paramount for accomplishing operational tasks and ensuring firefighter safety (Mendelson et al., 2023; Perroni et al., 2015). However, previous studies have focused more on firefighting equipment (Chae and Seung, 2022; Basodan et al., 2021; Bustos et al., 2021; Deng et al., 2018; Li et al., 2023; McQuerry et al., 2016; Morel et al., 2014; Rezazadeh and Torvi, 2011), strength and conditioning (Adetona et al., 2016; Chae, 2015; Cuenca-Lozano and Ramirez-Garcia, 2023; Igboanugo et al., 2021; McMorrow and Feairheller, 2022; Lentz et al., 2019; Meshkov et al., 2020), and injury prevention research (Carr-Pries et al., 2022; Eckard et al., 2018; Gallanter and Bozeman, 2002; Rabbitts et al., 2004; Sinnott et al., 2023), with relatively little study on firefighting rescue technology optimization and scientific strategies based on oxygen consumption, especially the experimental exploration of rescue strategies. In fact, firefighters need to decide which rescue techniques to use to accomplish which rescue task according to their fitness level to improve rescue efficiency and reduce the risk of safety and injury (Zhang et al., 2024). This scientific selection of rescue tasks and techniques based on the firefighters’ physical levels constitutes the optimal rescue strategy referred to in this study, namely, the study of the optimal configuration relationship between firefighter’s strength and conditioning, rescue tasks, and rescue techniques.

Therefore, this study aimed to use a separate three-factor mixed experimental design encompassing 3 (levels of strength and conditioning: excellent, good, and poor) × 3 (task loads: 30 kg large, 20 kg medium, and 10 kg small loads) × 3 (task load techniques: shoulder-carrying, bosom-carrying, and hand-carrying), to analyze the variations in oxygen consumption among firefighters of different fitness levels utilizing diverse rescue techniques to accomplish varied rescue tasks. It is hoped that the optimal configuration in rescue operations could be elucidated, providing a theoretical basis for improving rescue safety and proposing scientific rescue strategies.

## Methods

### Participants

Sixty healthy, professional male firefighters from the Guilin Fire and Rescue Detachment were randomly selected as participants. Their strength and conditioning level met the study design requirements (refer to the Description of sample data section) and were randomly selected as participants (age: 25.5 ± 3.7 years, height: 172.3 ± 6.7 cm, weight: 69.4 ± 5.9 kg, years of service: 4.9 ± 1.2 years). Inclusion criteria for firefighters included no history of injury or drug use in the past year. Before participation in the experiment, all participants provided written informed consent. The experiment adhered to the principles outlined in the Declaration of Helsinki and was approved by the Ethics Committee of Guangxi Normal University (20230419001).

### Experimental design and procedures

**FIGURE 1:**
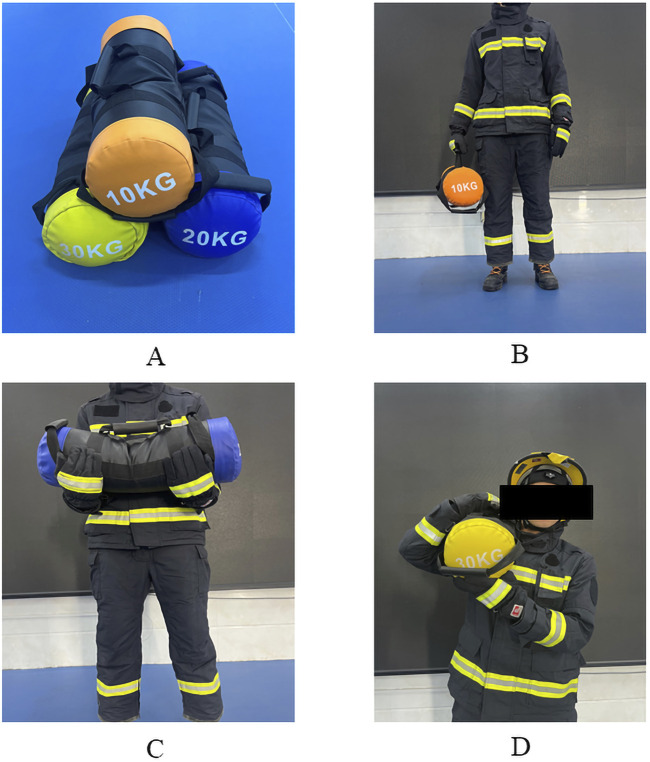
Task loads and load techniques. **(A)** Task loads; **(B)** Hand-carrying; **(C)** Bosom-carrying; **(D)** Shoulder-carrying.

In this study, three forms of exercise were designed to simulate firefighters’ fire rescue or training tasks: running ground (emulating firefighters’ 400-m material evacuation and rescue drill), running up and down stairs on three floors (each floor has 20 steps, each step has a height of 18 cm and a width of 28 cm, resulting in a total vertical climb of 3.6 m per floor) (emulating the weighted ascent to a building to fight a fire program). In addition, three rescue tasks were designed in this study with a load variable of 10, 20 and 30 kg sandbags (simulating training and fire rescue loads commonly used by firefighters), and three widely used load-carrying techniques: shoulder-carrying, bosom-carrying, and hand-carrying (Figure 1). Through experimental exploration, we aim to address the scientific inquiry of which strength and conditioning level firefighters are suited for which techniques to accomplish which loading tasks in a particular form of exercise. The experimental test comprises two main parts: the strength and conditioning level test and an experimental test of optimal rescue strategies.

### Strength and conditioning level test

The strength and conditioning level tests encompass strength performance (the upper and lower limb muscular strength) and speed performance evaluations. The primary criteria for assessing strength and speed performance levels are derived from the scoring standards outlined in the “Physical Fitness Training for Firefighters and Rescuers” (Editorial, 2020) and “Firefighting Physical Fitness Training (China Firefighters and Rescuers College Planning Teaching Material)” coursebooks (latest version) (Du, 2020). The strength performance test involves several steps: firstly, assessing the number of pull-ups and squats completed within 2 min. Subsequently, the upper and lower limb strength scores are calculated based on the respective scoring standards (Appendix 1). The combined average score of upper and lower limb strength exceeding 85 points is classified as excellent strength performance, while scores ranging from 70 to 85 points are considered good, and scores below 70 points are deemed poor. During the pull-up test, the participant faces the horizontal bar (RUNJOY RJ-JS-3253X, Sichuan, China), stands naturally, jumps to grasp the horizontal bar with an overhand grip, hands shoulder-width apart, and maintains a straight-armed hanging position. Once body movement stabilizes, both arms engage simultaneously to lift the body upward, ensuring no extraneous body motion during the ascent. A successful pull-up is counted when the chin over the upper edge of the horizontal bar, after which the participant returns to the initial straight-armed hanging position. During the squat test, participants were instructed to stand naturally with arms extended sideways and feet shoulder-width apart. While squatting, the knee joints were aligned with the direction of the feet, and the thighs were lowered until parallel to the ground or slightly below knee level. The speed performance test entails the following steps: initially, assessing the firefighter’s 100-m running performance. Subsequently, based on the scoring criteria, a score exceeding 85 is classified as excellent speed performance, scores ranging from 70 to 85 are considered good, and scores below 70 are deemed poor. The speed performance test is mainly timed with a stopwatch (CASIO HS-80TW-1, Tokyo, Japan) and requires participants to run a 100-m sprint at full speed. All participants undergo strength and speed performance tests 1 week before the experimental testing of optimal rescue strategies, and their results are meticulously recorded (Appendix 2).

## Experimental testing of optimal rescue strategies

**FIGURE 2:**
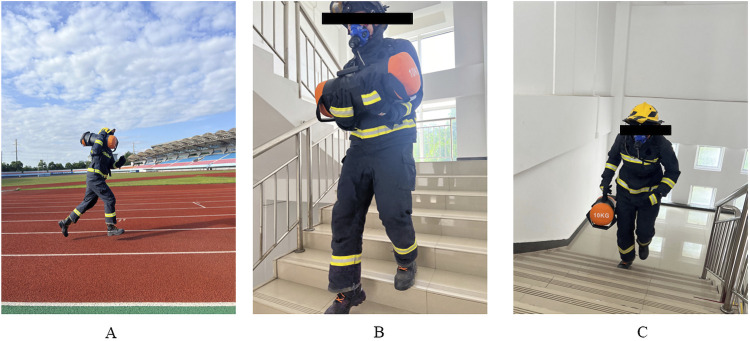
Optimal rescue strategies experimental test. **(A)** Running ground; **(B)** Running downstairs; **(C)** Running upstairs.

Wearing firefighting protective clothing and equipped with the wearable metabolic system (COSMED K5, Rome, Italy), firefighters undertook the experimental testing of optimal rescue strategies. The experiment comprised three exercise forms: running ground and up and down stairs. The standardized 400-m athletic field served as the experimental site for the running ground, while the same residential building was utilized for the running up and downstairs experiments. During the running ground experiment, firefighters used specified load techniques to carry designated loads to complete the 400-m running task. Similarly, during the running up or downstairs experiments, firefighters had to utilize specified load techniques to carry designated loads while completing the running up or downstairs on three-floor experiments (Figure 2). All tests required participants to run as fast as they could to complete a rescue task.

### Description of sample data

As this study employed a mixed factorial design across three specific exercise forms, each exercise form’s total number of task types amounted to 27 (3 strength and conditioning levels × 3 task loads × 3 task load techniques). However, each firefighter was permitted to complete experimental tests for up to nine tasks (3 task loads × 3 task load techniques). If firefighters of the same specified fitness level were to undertake all types of task tests, there would be a considerable risk of data distortion due to firefighter fatigue. Conversely, if the same firefighter were randomly assigned to complete only one task type, the number of participants would need to be significantly increased by a factor of 27 to maintain the same experimental effect, leading to escalated human resource costs for the experiment.

Therefore, in this study, three out of the nine task types formed by the two factors of task loads and load techniques were randomly selected for testing according to the firefighter’s strength performance level in a random order during the experimental tests of each exercise form (Appendix 3). This approach enabled nine participants with different strength and conditioning levels to complete 27 task tests. With the completion of 60 participants (six to seven rounds), 180 sample data for each form of exercise was generated, ensuring six to seven sample data for each task type. Moreover, due to the high degree of consistency between speed performance and strength performance, although speed performance was not considered when arranging the experimental tests in this study, the test results revealed that the sample data for each type of task from the speed performance perspective ranged from five to eight, aligning with the sample data requirements of the research design.

### Experimental outcomes and apparatus

The wearable metabolic system (COSMED K5) measured participants’ oxygen consumption from the beginning to the end of exercise using breath-by-breath mode, assessing physical performance by evaluating the flow, quantity, and volume of oxygen in exhaled breath. The system’s sensors analyze the exhaled gas sent through a turbine via a sampling line. Prior to each experiment, the flow turbine and gas analyzer were calibrated using a 3-L calibration syringe, gas, and regulator. Oxygen consumption values in this study were defined as the peak oxygen consumption from the beginning to the end of exercise, and the raw data were derived from COSMED K5 software. The oxygen consumption data were exported and then normalized according to body weight and task load, calculated as: oxygen consumption (mL/min/kg) = oxygen consumption (mL/min)/(bodyweight + task load) (kg), enabling fair comparisons across participants.

### Statistical analysis

All statistical analyses in this study were conducted using SPSS 26.0 software (IBMS, NY, USA). Given the repeated measures design, we employed a mixed-model ANOVA with participants as a random effect to account for individual differences. The model included strength and conditioning levels, task loads, and load techniques as fixed effects, and the covariance structure was selected based on model fit statistics (AIC and BIC). Bonferroni adjustment was used for *post hoc* pairwise comparisons. The statistical significance level was set at 0.05.

## Results

### Oxygen consumption of firefighters of different strength and conditioning levels performing different tasks while running ground

**FIGURE 3:**
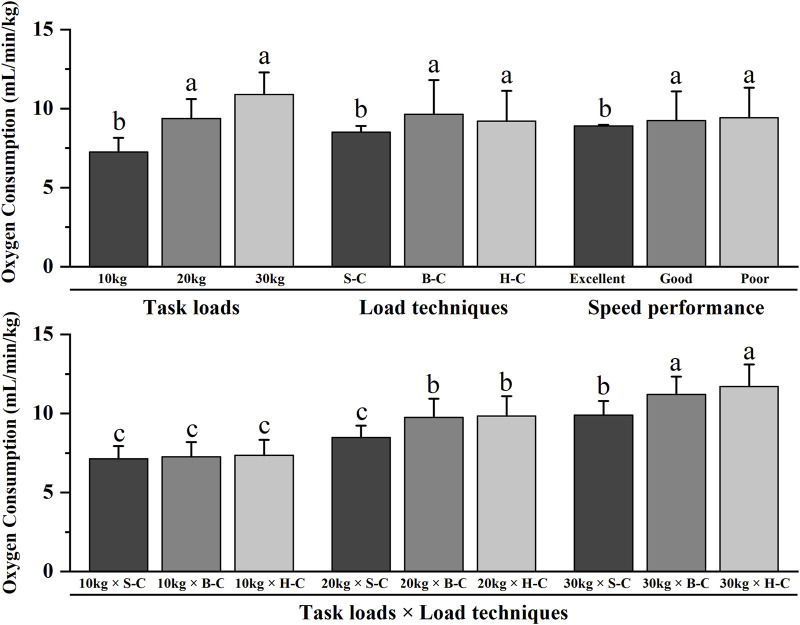
Interaction effects of oxygen consumption for running ground. Note: The figure only shows the results of *post hoc* pairwise comparisons for variables with significant differences in ANOVA; different letters between the two variables indicate significant differences (P < 0.05); data were expressed as mean ± standard deviation (Mean ± SD), and error bars indicate SD; S-C indicates shoulder-carrying; B-C indicates bosom-carrying; H-C indicates hand-carrying.

Table 1; Figure 3 revealed significant main effects for speed performance, task loads and load techniques (p < 0.05) but no significant main effect for strength performance (p > 0.05). Post hoc pairwise comparisons with Bonferroni correction showed that oxygen consumption was lower for firefighters with excellent speed performance, or when performing the 10 kg load task, or when using the shoulder-carrying techniques than other task types (p < 0.05). The interaction effects between task loads and load techniques were also significant (p < 0.05). Post hoc pairwise comparisons with Bonferroni correction showed that, for a 10 kg load task, the load techniques did not significantly affect oxygen consumption (p > 0.05). However, completion of both 20 and 30 kg load tasks using shoulder-carrying techniques resulted in lower oxygen consumption than other task types (p < 0.05).

**TABLE 1:** Summary of ANOVA results for oxygen consumption in running ground (N = 180, mL/min/kg).

Independent variables	F ratio	P-value	Independent variables	F ratio	p-value
Strength performance	2.110	0.125	Speed performance	3.501	**0.033**
Task loads	182.792	**<0.001**	Task loads	181.781	**<0.001**
Load techniques	19.887	**<0.001**	Load techniques	18.494	**<0.001**
Strength performance × Task loads	0.450	0.772	Speed performance × Task loads	1.094	0.362
Strength performance × Load techniques	1.852	0.121	Speed performance × Load techniques	0.763	0.551
Task loads × Load techniques	3.669	**0.007**	Task loads × Load techniques	3.866	**0.005**
Strength performance × Task loads × Load techniques	0.644	0.740	Speed performance × Task loads × Load techniques	1.044	0.406

Note: N = 180 represents the total number of observations, accounting for repeated measures across 60 participants. Left block: Main effects and interactions for strength performance. Right block: Main effects and interactions for speed performance. Bold values indicate significant differences in ANOVA results.

### Oxygen consumption of firefighters of different strength and conditioning levels performing different tasks while running upstairs

**FIGURE 4:**
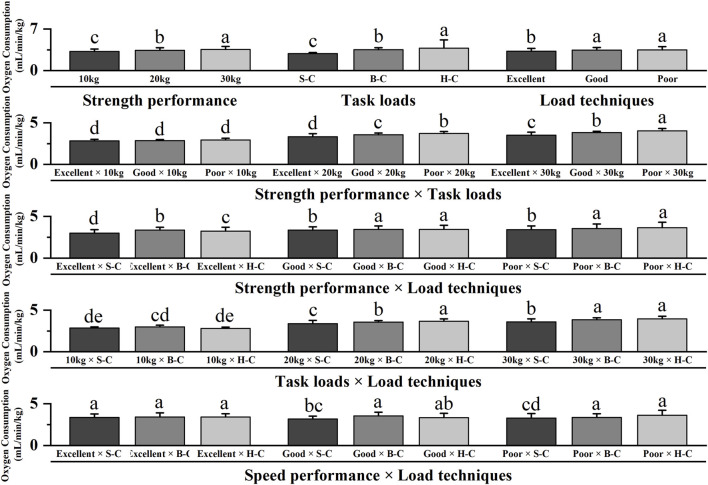
Interaction effects of oxygen consumption for running upstairs. Note: The figure only shows the results of *post hoc* pairwise comparisons for variables with significant differences in ANOVA; different letters between the two variables indicate significant differences (P < 0.05); data were expressed as mean ± standard deviation (Mean ± SD) and error bars indicate SD; S-C indicates shoulder-carrying; B-C indicates bosom-carrying; H-C indicates hand-carrying.

Table 2; Figure 4 showed significant main effects for strength performance, task loads and load techniques (p < 0.05) but no significant main effect for speed performance (p > 0.05). Post hoc pairwise comparisons with Bonferroni correction showed that firefighters with excellent strength performance or those tasked with carrying 10 kg loads or using shoulder-carrying techniques exhibited lower oxygen consumption than other task types (p < 0.05). The interaction effects were significant between strength performance and task loads, strength performance and load techniques, task loads and load techniques, speed performance and load techniques (p < 0.05). Post hoc pairwise comparisons with Bonferroni correction showed no discernible effect of strength performance on oxygen consumption when firefighters performed the 10 kg load task (p > 0.05). Firefighters with excellent strength performance levels demonstrated lower oxygen consumption than those with poor strength performance levels when executing the 20 and 30 kg load tasks (p < 0.05). Oxygen consumption of firefighters with excellent and poor strength performance levels employing shoulder-carrying load techniques was significantly lower than other task types (p < 0.05). Oxygen consumption during hand-carrying a 10 kg load task or shoulder-carried 20 and 30 kg load tasks was lower than other task types (p < 0.05). Oxygen consumption of firefighters with good and poor speed performance levels utilizing shoulder-carrying load techniques was less oxygen-intensive than other task types (p < 0.05).

**TABLE 2:** Summary of ANOVA results for oxygen consumption in running upstairs (N = 180, mL/min/kg).

Independent variables	F ratio	P-value	Independent variables	F ratio	p-value
Strength performance	37.166	**<0.001**	Speed performance	1.061	0.349
Task loads	271.973	**<0.001**	Task loads	195.165	**<0.001**
Load techniques	17.335	**<0.001**	Load techniques	9.585	**<0.001**
Strength performance × Task loads	4.879	**0.001**	Speed performance × Task loads	1.159	0.331
Strength performance × Load techniques	2.473	**0.047**	Speed performance × Load techniques	4.951	**0.001**
Task loads × Load techniques	4.734	**0.001**	Task loads × Load techniques	3.140	**0.016**
Strength performance × Task loads × Load techniques	1.168	0.323	Speed performance × Task loads × Load techniques	1.159	0.331

Note: N = 180 represents the total number of observations, accounting for repeated measures across 60 participants. Left block: Main effects and interactions for strength performance. Right block: Main effects and interactions for speed performance. Bold values indicate significant differences in ANOVA results.

### Oxygen consumption of firefighters of different strength and conditioning levels performing different tasks while running downstairs

**FIGURE 5:**
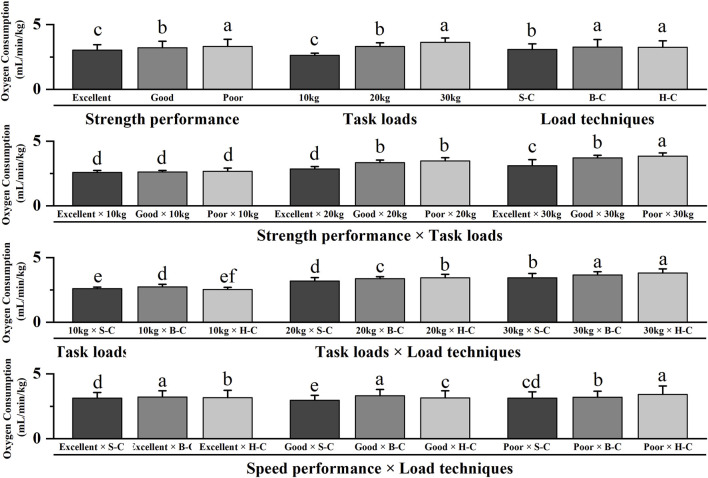
Interaction effects of oxygen consumption for running downstairs. Note: The figure only shows the results of *post hoc* pairwise comparisons for variables with significant differences in ANOVA; different letters between the two variables indicate significant differences (P < 0.05); data were expressed as mean ± standard deviation (Mean ± SD) and error bars indicate SD; S-C indicates shoulder-carrying; B-C indicates bosom-carrying; H-C indicates hand-carrying.

Table 3; Figure 5 showed significant main effects for strength performance, task loads and load techniques (p < 0.05) but no significant main effect for speed performance (p > 0.05). Post hoc pairwise comparisons with Bonferroni correction showed that firefighters with excellent strength performance levels, carrying 10 kg loads, or using shoulder-carrying techniques exhibited lower oxygen consumption than other task types (p < 0.05). The interaction effects between strength performance and task loads, task loads and load techniques, and speed performance and load techniques were significant (p < 0.05). Post hoc pairwise comparisons with Bonferroni correction showed that firefighters’ strength performance did not significantly impact oxygen consumption when completing a 10 kg load task (p > 0.05). When executing 20 and 30 kg load tasks, the oxygen consumption of firefighters with excellent strength performance levels was lower than other task types (p < 0.05). Oxygen consumption of firefighters performing 10 kg load tasks using hand-carried techniques and 20–30 kg load tasks using shoulder-carrying techniques was lower than other task types (p < 0.05). Firefighters with good and poor speed performance levels demonstrated significantly lower oxygen consumption with the shoulder-carrying technique than other task types (p < 0.05).

**TABLE 3:** Summary of ANOVA results for oxygen consumption in running downstairs (N = 180, mL/min/kg).

Independent variables	F ratio	P-value	Independent variables	F ratio	p-value
Strength performance	33.377	**<0.001**	Speed performance	2.924	0.057
Task loads	360.018	**<0.001**	Task loads	283.71	**<0.001**
Load techniques	16.851	**<0.001**	Load techniques	10.735	**<0.001**
Strength performance × Task loads	5.109	**0.001**	Speed performance × Task loads	1.608	0.175
Strength performance × Load techniques	0.911	0.459	Speed performance × Load techniques	5.149	**0.001**
Task loads × Load techniques	5.569	**<0.001**	Task loads × Load techniques	4.255	**0.003**
Strength performance × Task loads × Load techniques	0.848	0.562	Speed performance × Task loads × Load techniques	1.308	0.244

Note: N = 180 represents the total number of observations, accounting for repeated measures across 60 participants. Left block: Main effects and interactions for strength performance. Right block: Main effects and interactions for speed performance. Bold values indicate significant differences in ANOVA results.

## Discussion

This study aims to examine the impact of strength and conditioning levels, task loads, and load techniques on oxygen consumption during firefighter rescue tasks, aiming to identify optimal rescue strategies for the rescue process. The exercise pattern used to collect oxygen consumption in this study was very different from previous studies. Most previous studies have collected maximal oxygen consumption data by requiring participants to reach force exhaustion intensity under laboratory conditions. In contrast, the present study simulated a real fire and rescue task at an intensity far removed from the laboratory state of force exhaustion, and firefighters spent shorter periods of time performing rescue tasks than in the laboratory tests. As a result, the values in the present study are smaller compared to previous studies, but are more reflective of the true peak oxygen consumption of firefighters during rescue. The study findings revealed that, on the one hand, firefighters with excellent strength performance exhibited significantly lower oxygen consumption than their non-excellent performance when conducting the running up or downstairs rescue task. Conversely, firefighters with excellent speed performance demonstrated significantly lower oxygen consumption than non-excellent firefighters during the running ground rescue task. Additionally, firefighters carrying small loads or utilizing shoulder-carrying techniques to complete rescue tasks exhibited significantly lower oxygen consumption than other strength and conditioning levels, loads, or techniques. On the other hand, firefighters using the shoulder-carrying technique for medium and large loads rescue tasks displayed significantly lower oxygen consumption than those employing other load techniques. Furthermore, firefighters employing the hand-carrying technique for small load tasks during running up or downstairs rescues exhibited significantly lower oxygen consumption than those using other load techniques. Additionally, firefighters with excellent strength performance utilizing the shoulder-carrying technique for running upstairs during rescue tasks showed significantly lower oxygen consumption than those handling medium and large loads for running up or downstairs rescues, utilizing other load techniques.

### Independent effects of strength and conditioning levels, task loads, and load techniques on oxygen consumption

Firefighters with excellent strength are better suited for running up or downstairs rescue tasks, whereas those with excellent speed are more adept at running ground rescues. Strength and conditioning encompasses strength, speed, and endurance, significantly influencing sports performance (Bai et al., 2021; Shu et al., 2023; Zhang and Ren, 2022). Studies on stair walking have indicated that up or downstairs tasks entail greater resistance to movement and less stability than ground walking tasks (Song et al., 2016). Participants must exert greater force to overcome the effects of instability, thereby enhancing task performance efficiency. This could explain why firefighters with excellent strength performance exhibit significantly lower oxygen consumption during running up or downstairs rescue tasks compared to non-excellent level firefighters. Previous research on the relationship between firefighters’ strength performance and operational level has further confirmed this view: the strength level of firefighters is moderately correlated with the level of firefighting expertise, and the enhancement of strength can significantly improve the operational level of firefighters in the programs of “running upstairs” and “lifting and pulling loads” (Liu et al., 2022; Orr et al., 2021). However, unlike the up or downstairs task, the gravity potential energy change to be overcome in the running ground rescue task is lower (Winter 1987), and the key factor is to shorten the task execution time with a higher speed to improve the efficiency of the task, which may be the main reason why firefighters with excellent speed performance are more favourable to perform the running ground rescue task. In fact, different forms of exercise rescue tasks have specific influencing factors. The main factor affecting running up or downstairs rescue tasks is overcoming impaired motor performance due to the load, whereas the key aspect of running ground rescue tasks is the speed of body displacement. Therefore, from the perspective of firefighters’ conditional requirements to accomplish tasks, strength and speed emerge as the primary influencing factors for running up or downstairs and ground rescues, respectively. This explanation aligns with the findings of the present study based on the principles of sports biomechanics.

This study also found that firefighters who completed small-load tasks or used shoulder-carrying technique for rescues had lower oxygen consumption. Ensuring an adequate oxygen supply is vital for firefighters’ safety in rescue environments, often involving substantial smoke and toxic gases (Orysiak et al., 2022; Chen et al., 2022). Consequently, numerous previous studies have focused on enhancing oxygen utilization efficiency in air-breathing apparatus (Epstein et al., 1988; Li et al., 2003; Quesada et al., 2000). Previous studies have found that, on the one hand, task loads are a significant factor influencing oxygen consumption (Ruckstuhl et al., 2010). When the load exceeds 10% of body weight, respiratory amplitude and frequency significantly deepen and accelerate, leading to a sharp increase in oxygen consumption (Epstein et al., 1988; Li et al., 2003; Quesada et al., 2000). Increased loads during rescue operations prompt firefighters to intensify their movements, resulting in elevated heart rate, respiratory rate, oxygen consumption, and ventilation, rapidly depleting the air-breathing apparatus supply time and jeopardizing firefighter safety (Ras et al., 2024).

On the other hand, the loading technique is another crucial factor influencing oxygen consumption (Li et al., 2003). This study revealed that firefighters’ oxygen consumption was significantly lower when utilizing the shoulder-carrying technique to accomplish rescue tasks than other loading techniques. This phenomenon may stem from the alignment of the weight’s centre of gravity with the body’s central axis during shoulder-carrying, minimizing left-right and front-back moments and reducing ineffective work, thus conserving energy (Winter 1987). In contrast, bosom-carrying and hand-carrying techniques increase the body’s left-right and front-back moments, leading to heightened ineffective work and oxygen consumption. Additionally, from a human ergonomics perspective, shoulder-carrying engages more significant muscle groups of the lower limbs and trunk, enhancing the body’s work capacity and reducing oxygen consumption, while bosom-carrying and hand-carrying movements involve smaller muscle groups of the arms in rescue and load-carrying tasks (Winter 1987; Shim et al., 2013).

### Interactive effects of strength and conditioning levels, task loads, and load techniques on oxygen consumption

Firefighters are better suited to the shoulder-carrying technique when executing medium and large load tasks. In contrast, hand-carrying is more appropriate for running up or downstairs rescues involving small load tasks. Previous studies on the respiratory system’s response to loads and techniques have indicated that when the load is less than 10% of body weight, the loading technique does not significantly impact the respiratory system (Jokar et al., 2009). However, respiratory rate and air exchange volume notably increase when the load exceeds 15% of body weight (Li et al., 2003; Calabrese et al., 1998; Daubenspeck, 1981; Koga, 1990). While these studies have confirmed the influence of loads and techniques on rescue oxygen consumption, they have not proposed improved rescue strategies based on changes in oxygen consumption patterns, particularly lacking exploration of task-specific rescue strategies. Our study revealed that firefighters utilizing the shoulder-carrying technique to execute medium and large rescue tasks exhibit significantly lower oxygen consumption than other loads and techniques. This technique effectively reduces ineffective work, enhancing oxygen consumption efficiency—a biomechanical principle previously described. However, contrary to previous findings, we observed that firefighters consume less oxygen when employing the hand-carrying technique for small load running up or downstairs rescue tasks. Firefighters’ focus varies when performing tasks with different loads. The rescue task is relatively challenging when dealing with medium to large loads, with firefighters prioritizing task completion efficiency. The shoulder-carrying technique is favoured as it minimizes power dispersion and engages large muscle groups, ensuring successful task completion. Conversely, firefighters focus on task efficiency when handling tasks with small loads. Despite the increased involvement of small muscle groups and ineffective work associated with the hand-carrying technique, its portable advantage compensates for these drawbacks, enhancing task efficiency. This may explain why hand-carrying is more suitable for small-load task rescues involving running up or downstairs.

The study also revealed that firefighters with excellent strength levels were better suited for shoulder-carrying while running upstairs rescues than medium to large loads running up or downstairs rescues. In contrast, non-excellent firefighters at the speed level were more suited to the shoulder-carrying technique for running up or downstairs rescues. From the perspective of rescue tasks, running upstairs and downstairs requires higher strength levels from firefighters, albeit with differences in how strength is utilized. Running downstairs rescue primarily involves body posture control, while running upstairs rescue focuses on overcoming the loads. Compared to hand-carrying and bosom-carrying techniques, the shoulder-carrying technique ensures that the load’s centre of gravity is closer to the human body’s central axis, facilitating efficient force transfer and reducing ineffective work. This likely explains why firefighters with good strength levels are more suitable for adopting the shoulder-carrying technique for running upstairs than downstairs. Loads are another crucial factor influencing oxygen consumption in strength-based tasks. Previous studies have indicated that the advantage of excellent strength levels in load transportation capacity becomes apparent only when the load reaches a certain weight (Li et al., 2003; Calabrese et al., 1998; Daubenspeck, 1981; Koga, 1990; Brack et al., 1997; Hagins and Lamberg, 2006). Our findings align with this, highlighting that firefighters with excellent strength levels have lower oxygen consumption when handling medium to large loads.

Interestingly, the study also observed that firefighters with excellent strength levels consumed less oxygen when employing the shoulder-carrying technique in downstairs rescues of medium to large loads. This is the same principle as the biomechanical principle, suggesting that the shoulder-carrying technique is more suitable for firefighters with excellent strength levels during running upstairs rescues, disregarding load variables. Postural control costs increase, and movement stability becomes a major challenge when performing large loads while running downstairs rescues. Higher strength levels can enhance movement stability, reduce inefficient work, and reduce oxygen consumption. Furthermore, the findings suggest that non-excellent firefighters at the speed level are better suited for adopting the shoulder-carrying technique for running up or downstairs rescues. While shoulder-carrying techniques can reduce ineffective work and oxygen consumption during up or downstairs rescues, firefighters with excellent speed levels do not exhibit oxygen utilization advantages contrary to expectations. This could be attributed to the decrease in movement stability accompanying increased speed, leading to elevated ineffective work and oxygen consumption during rescue operations. Thus, firefighters should prioritize stability over speed to reduce oxygen consumption during up or downstairs rescues. Future research could design controlled experiments to investigate firefighters’ oxygen consumption patterns during up or downstairs rescues based on speed level choices between “fast” and “steady.”

### Study limitations and perspective

While our experimental design and testing process were meticulously controlled, several limitations warrant consideration for future research. Firstly, the study was conducted in a simulated rescue environment rather than a fireground setting. As such, questions remain regarding the extent to which participants’ performance in our study accurately reflects real-world fireground scenarios. Subsequent research should aim to validate our findings by comparing them with outcomes obtained from authentic fireground tests. Secondly, our investigations of firefighters’ strength and conditioning levels explored only strength and speed performance, and did not explore capabilities such as aerobic and agility. Investigations of tasks focused primarily on variations in load weight size, with less emphasis on the influence of load shape when executing different rescue task configurations. Future studies should seek to expand upon our findings by exploring these additional factors. Thirdly, our evaluation criteria predominantly centred on assessing oxygen consumption to gauge the rationality and efficacy of rescue strategies. However, alternative objective metrics exist, including time, effectiveness, and biomechanical parameters (such as joint moments, angles, and stiffness). Future research should investigate the impact of these alternative evaluation criteria to provide a comprehensive assessment and identify the optimal firefighting rescue strategy. Finally, due to the fact that this study utilized a mixed-factorial design with a large number of experimental groups, which was a great challenge for participant recruitment, the overall study sample size was slightly insufficient despite the use of a clever experimental design that ensured sample distribution. Future studies could expand the sample size to validate this study’s findings further.

## Conclusions

Firefighters with excellent strength performance are better equipped for rescue tasks involving running up or down stairs, while those with excellent speed performance are more suited for tasks on running ground. Additionally, firefighters consume less oxygen when performing tasks with smaller loads or using shoulder-carrying techniques. It is recommended that firefighters utilize shoulder-carrying for medium to large load tasks and hand-carrying for smaller loads during stair runs. Those with strong strength performance should prioritize shoulder-carrying for stair runs and medium to large loads. Conversely, firefighters with less than excellent speed performance are more suited to shoulder-carrying during stair runs.

## Data Availability

The original contributions presented in the study are included in the article/supplementary material, further inquiries can be directed to the corresponding author/s.
